# Do COVID-19 Infectious Disease Models Incorporate the Social Determinants of Health? A Systematic Review

**DOI:** 10.3389/phrs.2024.1607057

**Published:** 2024-10-10

**Authors:** Ava A. John-Baptiste, Marc Moulin, Zhe Li, Darren Hamilton, Gabrielle Crichlow, Daniel Eisenkraft Klein, Feben W. Alemu, Lina Ghattas, Kathryn McDonald, Miqdad Asaria, Cameron Sharpe, Ekta Pandya, Nasheed Moqueet, David Champredon, Seyed M. Moghadas, Lisa A. Cooper, Andrew Pinto, Saverio Stranges, Margaret J. Haworth-Brockman, Alison Galvani, Shehzad Ali

**Affiliations:** ^1^ Department of Epidemiology and Biostatistics, Schulich School of Medicine and Dentistry, Western University, London, ON, Canada; ^2^ Department of Anesthesia and Perioperative Medicine, Schulich School of Medicine and Dentistry, Western University, London, ON, Canada; ^3^ Centre for Medical Evidence, Decision Integrity and Clinical Impact, Schulich School of Medicine and Dentistry, Western University, London, ON, Canada; ^4^ Lawson Health Research Institute, London, ON, Canada; ^5^ Schulich Interfaculty Program in Public Health, Schulich School of Medicine and Dentistry, Western University, London, ON, Canada; ^6^ Health Sciences Library, London Health Sciences Centre, London, ON, Canada; ^7^ University of Ottawa Heart Institute, Ottawa, ON, Canada; ^8^ School of Health Studies, Faculty of Health Sciences, Western University, London, ON, Canada; ^9^ Dalla Lana School of Public Health, University of Toronto, Toronto, ON, Canada; ^10^ Johns Hopkins Center for Health Equity, Johns Hopkins University, Baltimore, MD, United States; ^11^ Department of Health Policy, London School of Economics and Political Science, London, United Kingdom; ^12^ Public Health Agency of Canada (PHAC), Ottawa, ON, Canada; ^13^ Department of Mathematics and Statistics, Faculty of Science, York University, Toronto, ON, Canada; ^14^ Department of Family and Community Medicine, St. Michael’s Hospital, Toronto, ON, Canada; ^15^ Department of Family and Community Medicine, Faculty of Medicine, University of Toronto, Toronto, ON, Canada; ^16^ Li Ka Shing Knowledge Institute, St Michael’s Hospital, Toronto, ON, Canada; ^17^ Institute of Health Policy Management and Evaluation, Dalla Lana School of Public Health, University of Toronto, Toronto, ON, Canada; ^18^ Department of Clinical Medicine and Surgery, University of Naples Federico II, Naples, Italy; ^19^ National Collaborating Centre for Infectious Diseases, Rady Faculty of Health Sciences, University of Manitoba, Winnipeg, MB, Canada; ^20^ School of Public Health, Yale University, New Haven, CT, United States; ^21^ Department of Health Sciences, University of York, University of Manitoba, York, United Kingdom; ^22^ World Health Organization Collaborating Centre for Knowledge Translation and Health Technology Assessment in Health Equity, Ottawa, ON, Canada; ^23^ Department of Psychology, Macquarie University, Sydney, NSW, Australia

**Keywords:** COVID-19, infectious disease models, social determinants of health, public health, model validity

## Abstract

**Objectives:**

To identify COVID-19 infectious disease models that accounted for social determinants of health (SDH).

**Methods:**

We searched MEDLINE, EMBASE, Cochrane Library, medRxiv, and the Web of Science from December 2019 to August 2020. We included mathematical modelling studies focused on humans investigating COVID-19 impact and including at least one SDH. We abstracted study characteristics (e.g., country, model type, social determinants of health) and appraised study quality using best practices guidelines.

**Results:**

83 studies were included. Most pertained to multiple countries (n = 15), the United States (n = 12), or China (n = 7). Most models were compartmental (n = 45) and agent-based (n = 7). Age was the most incorporated SDH (n = 74), followed by gender (n = 15), race/ethnicity (n = 7) and remote/rural location (n = 6). Most models reflected the dynamic nature of infectious disease spread (n = 51, 61%) but few reported on internal (n = 10, 12%) or external (n = 31, 37%) model validation.

**Conclusion:**

Few models published early in the pandemic accounted for SDH other than age. Neglect of SDH in mathematical models of disease spread may result in foregone opportunities to understand differential impacts of the pandemic and to assess targeted interventions.

**Systematic Review Registration:**

[https://www.crd.york.ac.uk/prospero/display_record.php?ID=CRD42020207706], PROSPERO, CRD42020207706.

## Introduction

Infectious disease models are tools that help researchers simulate real-world possibilities in a virtual environment and inform public health policy decisions. Models use mathematical equations to anticipate the future course of an outbreak, aid public health planning, and support disease control efforts. The models describe “how infectious diseases progress in a given population depending on existing and counterfactual conditions/measures and a disease’s characteristic (e.g., transmission rate, incubation, asymptomatic case).” [1] To build these models, analysts synthesize information from a variety of sources, including epidemiological surveillance data, the published literature, and expert opinion. The validity of the models can be tested by changing the input parameters within plausible ranges, also known as uncertainty analysis, and by comparing the model predictions to epidemiological surveillance data.

Infectious disease models can be grouped into three types: compartmental, agent-based, and statistical models [1]. Each differs in the way the modelled population is conceptualized. Compartmental models divide the simulated population into groups, and changes in disease incidence over time are a function of interactions amongst the groups. In the simplest compartmental models, simulated groups are defined only by infection status (e.g., susceptible, infected), however the number of groups expands greatly if modelers wish to account for additional characteristics (for example, age group and gender). Agent-based models assign characteristics to simulated individuals, rather than groups, and changes in disease incidence over time are a function of interactions amongst simulated individuals. Statistical models predict infection risks in a simulated population without explicitly modelling interactions. Statistical models take different forms. Curve fitting models, sometimes called auto-regressive models, fit epidemic curves to historical data and extrapolate the curves to simulate and predict future incidence. Other statistical models use data on population or cohort characteristics to simulate and predict infection risk. Non-communicable disease models can be used to model COVID-19 risk or health system impacts for a simulated population, as a function of cohort or population characteristics [2]. Models have a range of complexities and there are often tradeoffs between developing tractable models that can inform policy decisions in a timely manner and accurately reflecting important sources of heterogeneity, such as social determinants of health.

The World Health Organization defines the social determinants of health as the “non-medical factors that influence health outcomes” and “the conditions in which people are born, grow, work, live, and age, and the wider set of forces and systems shaping the conditions of daily life,” which include “economic policies and systems, development agendas, social norms, social policies and political systems” [3]. The WHO social determinants of health include income and social protection, education, unemployment, job insecurity, working life conditions, food insecurity, housing, basic amenities and the environment, early childhood development, social inclusion and non-discrimination, structural conflict, and access to affordable health services of decent quality [3]. Geography can also be considered a social determinant of health, when the aforementioned factors differ spatially, by neighborhood, county, country, or region [4, 5]. Gender, as a social construct, is a non-medical factor associated with the availability of care and is thus a social determinant of health [6, 7]. Sex, as a biological attribute is related to disease susceptibility and progression. We will report on gender as a social determinant, recognizing that modelling studies may have reported on sex, either conflating gender and sex in the conceptualization of the model, or intending to conceptualize sex differences in COVID-19 susceptibility and outcomes.

Early in the pandemic, empirical evidence identified significant variation in the epidemiology and impact of COVID-19 as a function of the social determinants of health. Counties in the United States with a higher proportion of African-Americans demonstrated significantly elevated rates of COVID-19 infection and death compared to other counties, even after controlling for demographic, clinical, social and environmental factors [8]. In England, Black and Asian ethnic minorities experienced elevated rates of COVID-19 diagnoses, compared to White residents [9]. Essential workers disproportionately came from low socioeconomic backgrounds (e.g., meat processing workers, temporary/migrant farm workers) and faced elevated COVID-19 occupational health and safety hazards [10]. These disparities are a symptom of deeper societal and health system inequities, including disproportionate exposure to infection risk, prevalence of comorbidities, and inequitable access to testing and treatment [11].

The goals of this systematic review of the literature were to identify COVID-19 infectious disease models that accounted for social determinants of health and characterize the extent to which COVID-19 models incorporate the social determinants of health. The systematic review is focused on models published early in the pandemic (during a period corresponding to the first two waves). Models published early in the pandemic reflect at least in part the preparedness of the modelling community to support responses to an emerging infectious disease threat. The early pandemic period represented an opportunity to reduce morbidity and mortality of COVID-19 impact through policies and interventions that were well informed and thoroughly evaluated. Incorporating social determinants of health into COVID-19 models can better support the development and appraisal of policies and targeted interventions than models based on an average population approach [12]. Including social determinants of health in models is also necessary to understand both the equity and efficiency of COVID-19 control efforts.

## Methods

We conducted a systematic review of the literature according to the Preferred Reporting Items for Systematic Reviews and Meta-Analyses (PRISMA) guidelines [13]. The protocol is registered in the International Prospective Register of Systematic Reviews (PROSPERO) (Registration number CRD42020207706), and published in the peer-reviewed literature [14].

### Search Strategy and Eligibility Criteria

We searched MEDLINE, EMBASE, Cochrane Library, medRxiv, and the Web of Science databases to identify all COVID-19 modelling studies published from December 2019 to August 14, 2020. Studies were deemed eligible if they: 1) focused on SARS-CoV-2 in a human population, 2) were a modelling study, 3) investigated one of the following outcomes: COVID-19 disease-related outcomes, non-COVID-19 disease-related outcomes (i.e., indirect impacts on other health conditions), impacts on health services, impact of policies or interventions on COVID-19 outcomes or its societal impact, and 4) included at least one social determinant of health. We excluded studies that focused on: 1) within-host biological studies, 2) non-human SARS-CoV-2 studies, 3) phylogenic/genetic studies, 4) environmental or meteorological studies without health impact analysis on humans, 5) epidemiological or statistical analyses without a model, 6) economic analyses without a model, and 7) COVID-19 infectious disease models that did not include a social determinant of health. A search strategy is provided in Supplementary File S1.

We made one amendment to the original systematic review protocol [14]. The original protocol included plans to assess social determinants of health in preprint models. Although our search strategy targeted preprints, we amended the protocol to exclude preprint models at the screening stage. Due to the poor quality of reporting in preprint models and the large volume of preprint models, we judged that the insights gleaned would not be worth the significant investment in systematic review resources that would be required to abstract data from preprint models.

### Review Process

Using Covidence Software (v204501fd4fa9), duplicate citations were removed. Titles and abstracts were screened independently in duplicate (GC, FWA, LG, EP, CS) to identify studies that potentially met the inclusion criteria outlined above. Conflicts were resolved by a third independent reviewer (DEK, NM, MM, ZL). Full text articles were retrieved and independently assessed for eligibility in duplicate (GC, LG, FWA, EP, CS). Disagreements were resolved by a third reviewer (DEK, NM, MM, ZL).

### Data Extraction and Quality Assessment

Data were extracted by one reviewer (GC, LG, FWA, EP, CS) and confirmed by a second (AJB, ZL). Data extracted from studies included: author, publication year, country, target population, modelling goals, type of model, social determinants of health, and approach to incorporating socioeconomic factors. We abstracted model type in the following categories: compartmental models, simulation models, multi-state life table models, comparative risk assessment models, regression models, autoregressive models, network or agent-based simulations, and other model types. Model type categories were based on systems for infectious disease models and non-communicable disease models, which distinguish between statistical models and non-statistical models, modelling of individuals or groups, and whether or not models incorporate interactions amongst modelled entities [1, 2]. Where relevant, we abstracted the interventions or policies appraised by the model. When multiple intervention options that differed only by the level of intensity or assumptions about adherence were compared, we did not record them as separate interventions. We assessed whether model results were reported by social determinants (e.g., age or race/ethnicity), and whether interventions were targeted according to social determinants, or varied by social determinants. We also recorded whether the goal of the modelling exercise was to evaluate the impact of COVID-19 by social determinants.

We developed a checklist for assessing the quality of infectious disease models (Supplementary File S2) based on the principles of best practices for models [15, 16]. Quality appraisal focused on justification of the model structure and assumptions, conduct of sensitivity analysis, internal validation (i.e., verifying that model equations have been accurately implemented and fit data used to develop the model, often referred to as model calibration), external validation (i.e., comparing model outputs to data that were not used to develop the model), completeness and transparency in model reporting, including the provision of model equations, code and initial values. Model appraisal was conducted by one reviewer (GC, LG, FWA, EP) and confirmed by a second (AJB, ZL), and was based on the analyses reported by the study authors. For example, study authors may have conducted internal validation of models, but if this was not reported in the manuscript, it was assumed that it was not performed.

## Results

**FIGURE 1:**
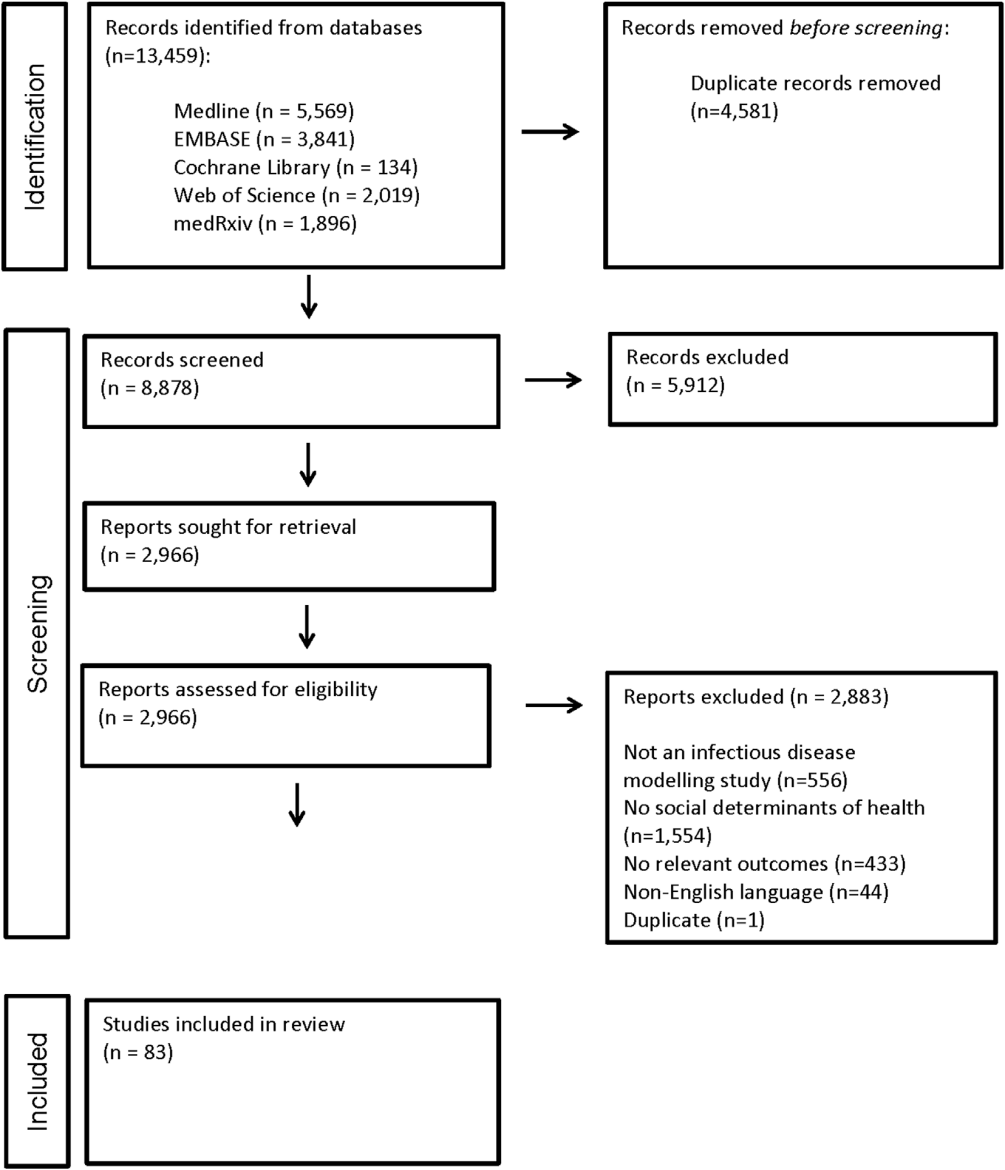
PRISMA flow diagram (Worldwide. 2020).

A total of 8,878 unique citations were identified and screened (Figure 1). Of these, 2,966 full-text manuscripts were assessed for eligibility (Figure 1). Only 83 studies were included in the review, reflecting that the vast majority of potentially eligible COVID-19 modelling studies did not include social determinants (Figure 1).

**FIGURE 2:**
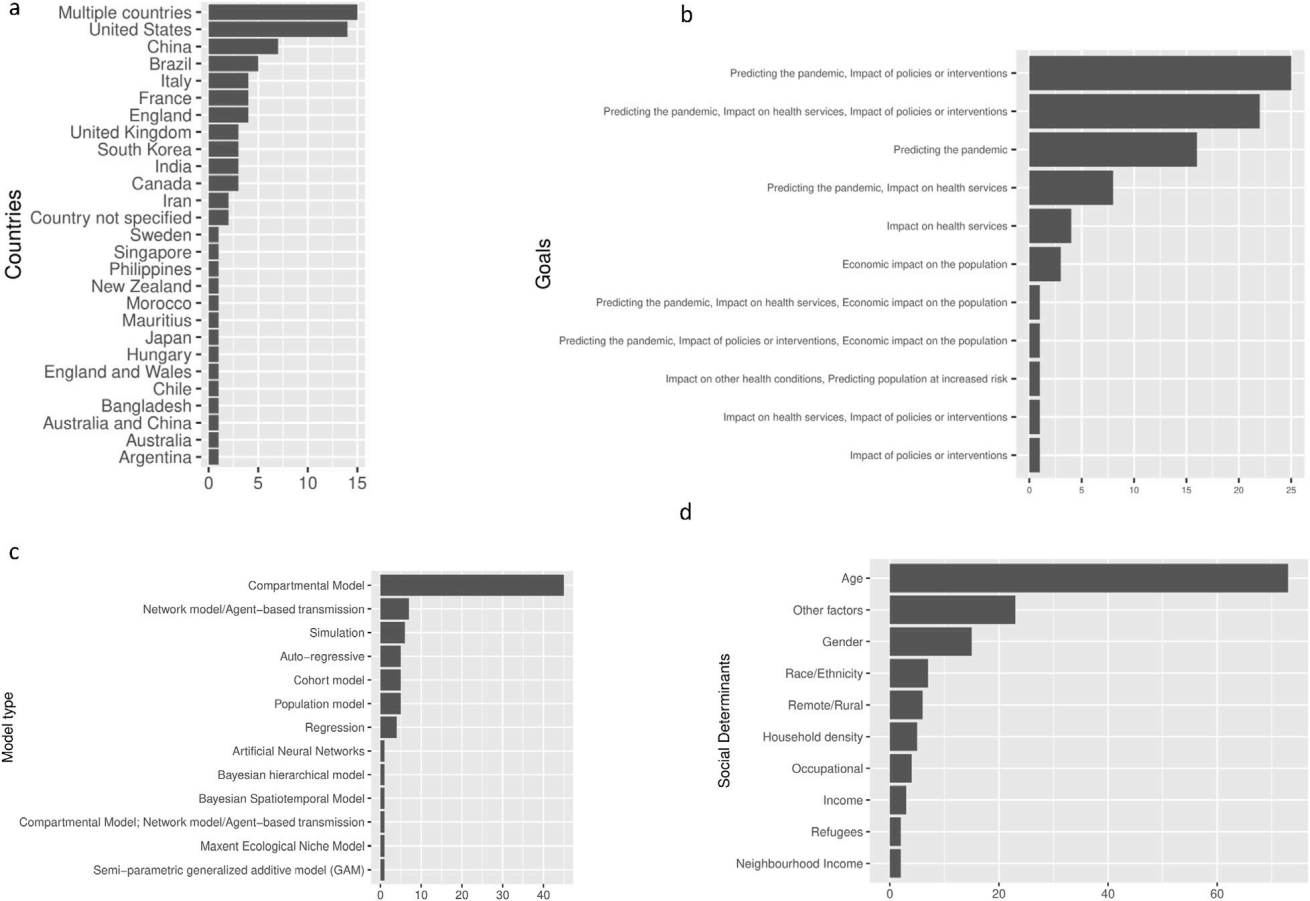
Characteristics of included studies (Worldwide. 2020). **(A)**: Distribution of countries in the included studies (Worldwide. 2020). **(B)**: Goals addressed by the studies (Worldwide. 2020). **(C)**: Distribution of model types (Worldwide. 2020). **(D)**: Social determinants included in the studies (Worldwide. 2020).

Of the studies included, most models pertained to multiple countries (n = 15, 18%), the United States (n = 12, 14%), or China (n = 7, 8%) (Figure 2A). The majority addressed two or more goals: (a) 24 studies focused on predicting the pandemic and assessing the impact of policies or interventions; (b) 21 studies focused on predicting the pandemic, assessing the impact on health services, and assessing the impact of policies or interventions; and (c) 17 studies focused only on predicting the pandemic (Figure 2B). The most common model types that incorporated interaction amongst modelled entities, included compartmental (n = 43, 52%) and agent-based (n = 7, 8%) models. Regression models, including those with regression analysis of area-level data (n = 6, 7%) and comparative risk assessment models (n = 6, 7%), were the next most common, followed by simulation models (n = 5, 6%), models with auto-regressive curve fitting (n = 5, 6%), and multi-state life table models (n = 5, 6%). Other model types included one of the following: Maxent ecological niche model, Bayesian hierarchical model, Bayesian spatiotemporal model, artificial neural networks, and a semi-parametric generalized additive model (Figure 2C).

The most common social determinant included in the models was age (n = 74, 89%). Of the included studies, 36 (43%) incorporated non-age social determinants. The most common non-age social determinants included in the models were gender (n = 13, 16%), race/ethnicity (n = 7, 8%), remote/rural location (n = 6, 7%), and household density/size (n = 5, 6%) (Figure 2D). Overall, 26% of compartmental models and 43% of agent-based models incorporated non-age social determinants. All regression analyses, 83% of comparative risk assessment models, 60% of auto-regressive models and 60% of simulation models included non-age social determinants (Supplementary Table S1). Thus, regression analysis and comparative risk assessment models were more likely to include non-age social determinants.

The approach to incorporating social determinants differed by model type. Stratification was a common approach to incorporating social determinants in compartmental models, comparative risk assessment models and multi-state life table models. Compartmental models stratified the modelled population by the social factor, most commonly by age group, using age-group specific contact rates amongst age-groups [17–19]. Compartmental models also stratified the population according to income [20], gender [21, 22], household density [23] and occupational factors [24, 25]. For example, Laurio Dizon et al. modelled a separate compartment for those required to work during shutdowns (mobile workers) to capture the risk of COVID-19 spread associated with occupational factors [25]. In comparative risk assessment models, risks specific to strata defined by gender [26, 27], household density [26], and rural/urban divides [28], were used to predict COVID-19 related outcomes. Multi-state life table models also incorporated risk-specific strata, by age group, to estimate the present value of lives lost [29], and differences in the negative impacts of COVID-19 control policies on HIV-related mortality [30].

In auto-regressive modelling studies, social determinants were incorporated by fitting curves to incidence data from regions with varying levels of social determinants such as population density [31], gender ratio [32], and distance to public spaces [33]. Auto-regressive modelling studies also incorporated social determinants as a time-varying covariate reflecting changes in the age distribution of those infected with COVID-19 over time [34, 35]. Regression analyses that were not auto-regressive used area-level data to estimate the association between social determinants and COVID-19 risk at the ecological level [36–40]. For example, in one study, COVID-19 risk was modelled as a function of regional characteristics such as the percentage of black individuals in each state [37], while population density, mobility data, the number of restaurants, and the number of supermarkets were used in another [36]. In agent-based models and simulation models social determinants were person-level characteristics signifying different risk levels [41, 42].

Model type was also associated with modelling goals. All agent-based models (7/7, 100% and most compartmental models (37/45, 82%) assessed the impact of policies or interventions, whereas few regression (1/5, 20%) and auto-regressive models (1/4, 25%) assessed policies or interventions. Thus, the model types more likely to incorporate a broader range of social determinants were less frequently used to appraise the impact of policies or interventions.

Even though all the included studies incorporated at least one social factor into the COVID-19 model, a significant proportion did not report model findings according to social factor (n = 29, 35%). We identified some studies that assessed interventions that were either targeted by social factor or varied by social factor (n = 22, 27%). For example, Jamieson-Lane and colleagues evaluated targeted sequestration of older age groups [43], and Davies et al. examined school closures targeting individuals under age 20 [44].

Twelve studies (12%) explicitly stated that the goal of the modelling was to examine the impact of social determinants. Studies estimated age-standardized infection rates [20], age-specific fatality rates [45], rates of infection by race [37], intergenerational impacts of COVID-19 infection [35], and how variation in co-residence patterns across countries is associated with susceptibility to COVID-19 outbreaks [45]. Others compared the predictions of models with and without age structure [17, 31, 46–49], or with different assumptions about age distribution [50]. No studies examined the effect of including other social determinants on internal or external model validation.

The study by Koo et al. included the largest number of social determinants, incorporating parameters for age, gender, race/ethnicity, immigration status, income, occupational factors, employment status, hours worked, industry, religion, marital status, number of children, education, housing type, enrollment in the national service health insurance, transportation mode, transportation time, and mobility status into an agent-based model [51]. Thus, this study included both household-level and individual-level factors in an agent-based simulation.

### Quality Appraisal

Appraisal of model quality revealed that most models included in the systematic review reflected the dynamic nature of infectious disease spread (n = 51, 61%) in which the risk of infection is a function of the number of infected individuals over time. Most authors provided justification of the model structure (n = 53, 64%), clearly specified model assumptions (n = 73, 88%), performed sensitivity analysis on key input parameters (n = 46, 55%), and conducted uncertainty analysis on key structural assumptions related to infectious disease spread (n = 50, 60%). Few studies considered thresholds for epidemic spread or extinction related to COVID-19 (n = 14, 17%), few reported performing internal model validation (n = 10, 12%), and fewer than half reported performing external validation of models (n = 31, 37%). Many study authors provided model equations (n = 41, 49%), however relatively few provided the code to reproduce the analysis (n = 23, 28%).

## Discussion

In our systematic review of the literature on COVID-19 infectious disease models, we identified 83 studies that included social determinants of health, representing fewer than 3% of the full-text articles screened. Age was the most common social factor, incorporated into 89% of included studies. Some social determinants known to play an important role in COVID-19 spread were not represented well in models published early in the pandemic. We found only a few models that incorporated occupational factors despite the knowledge that essential workers were at increased risk of contracting COVID-19. We also found no models addressing substance users, sexual minorities, or incarcerated persons, which were particularly vulnerable populations.

Our study represents a unique contribution to the literature as we are unaware of any other systematic reviews focused on incorporation of social determinants of health in COVID-19 models. Gerlee et al performed a systematic review to identify Swedish COVID-19 modelling studies from the early pandemic period [52]. The goal of this research effort was to appraise the predictive accuracy of the models, and no information on inclusion of social determinants of health was abstracted [52]. Kimani et al conducted a systematic review identifying 74 COVID-19 modelling studies pertaining to the Africa region [53]. While no information on inclusion of social determinants of health was abstracted in this study, the finding that only 7% of the models were calibrated using demographic data, suggests that few of the identified models incorporated social determinants of health [53].

Infectious disease modelling guidelines stress the importance of reflecting significant sources of heterogeneity due to differences in risk behaviours, morbidity, mortality, and the rate of uptake of interventions to accurately reflect transmission dynamics and improve model accuracy and precision [1, 12, 54–56]. Social determinants represent potentially important sources of heterogeneity as COVID-19 risks vary for important groups. Incorporating social determinants into models may be hampered by inadequate data. A systematic review of peer-reviewed quantitative studies that was published early in the pandemic identified estimates of COVID-19 risk specific to sub-groups defined by race, ethnicity and socioeconomic deprivation, but limited evidence was found on other key determinants, including occupation, educational attainment, housing status and food security [57]. Quantitative estimates, even when available, may not be in the optimal format. Early in the pandemic, quantitative estimates of differential impacts were provided at the ecological level, pertaining to regions rather than individuals [8]. One of the benefits of modelling is that limited data, while not optimal, can still inform parameter estimates. For example, area-level estimates may still be useful for model validation even when individual data are unavailable.

Modellers are often required to make tradeoffs between model complexity and feasibility. Model types that provide the flexibility to facilitate incorporation of social determinants, such as agent-based models, require more computational power. Model types requiring less computing power limit flexibility for incorporating social determinants into models. For example, we found compartmental models, which are less computationally intensive, less likely to include non-age social determinants. On the other hand, agent-based models incorporated multiple social determinants. Implementation and analysis of agent-based models requires greater investments in resources, particularly computational power. Statistical models, such as regression analyses and auto-regressive approaches, were more likely to include social determinants, but many of these studies used an ecological approach. Applying an ecological approach to social determinants in regression models (e.g., neighbourhood deprivation, racial/ethnic concentration) is useful for using regional characteristics to identify pandemic hotspots, but does not provide the flexibility to evaluate the impact of policies or interventions at the individual-level. Models that do not incorporate social determinants may be adequate to inform general public health policies. We found some studies that assessed improvements in model validity derived from including age strata, however, none examined the effect of including other social determinants on model validity. The benefits of including social determinants in COVID-19 models were largely unexplored early in the pandemic.

Infectious disease models are one input into decision making processes. Other types of research evidence may have influenced the devising and implementation of COVID-19 control policies targeting vulnerable populations. Epidemiological analyses, news media reports, or advocacy efforts may have provided the impetus for addressing COVID-19 and targeting certain vulnerable populations. For example, evidence that incidence was highest in United States counties with a high percentage of African American residents, may have promoted policies targeting this high-risk group even if African American ethnicity was not accounted for in published models. In the absence of data, analysts, policymakers, and advocates may have promoted consideration of social determinants in decision-making processes. However, neglecting to represent social determinants in models means the opportunity to apply the power of mathematical modelling to forecast the potential effectiveness of targeted policies was foregone. Targeting can potentially be a more efficient mechanism of infectious disease control. For example, handing out masks to individuals in a low-income neighbourhood early in the pandemic might have reduced morbidity and mortality in low-income communities, and may have also decreased COVID-19 spread overall.

Decision makers are interested in models that address the social determinants of health. A qualitative investigation of North Carolina state policymakers revealed a desire for COVID-19 models “to show disease spread within subpopulations, including by race/ethnicity, to understand and predict how groups experiencing a higher burden of disease shifted over time” [58]. Making efforts to incorporate social determinants of health into mathematical models can be considered an ethical imperative to overcome historical injustices that lead to both the disparities in health outcomes associated with social determinants and the lack of available data to inform social determinants of health in mathematical models [59].

### Limitations

Our study has several limitations. Published COVID-19 models may not be representative of all models. Modellers directly informing government decision-makers may not have published model findings. A systematic review of the grey literature to identify and appraise non-peer-reviewed models was beyond the scope of this study. However, our personal experience of policy models in Canada, the United States, and the United Kingdom, suggests these models infrequently included non-age determinants of health. Our systematic review covers a period early in the pandemic, corresponding to the first two waves. Because models incorporating social determinants of health are more complex, it is possible that these more complex models took more time to develop, validate and publish. As a result, the models published early in the pandemic could reflect a subset of simpler COVID-19 models that did not incorporate social determinants of health. It is also possible that modelers updated extant models to incorporate social determinants. More recently published models may have incorporated social determinants at a higher rate, particularly as attention to social determinants increased. However, making changes to models requires allocating resources to evidence synthesis, model programming and model validation. As the pandemic evolved, modelers adapted models to reflect new variants and to evaluate changing public health policies (e.g., vaccination and easing of public health measures). Adapting models to keep pace with changes in the pandemic may have taken priority over adapting models to incorporate social determinants, as this change would have been difficult to accomplish amidst competing priorities. Therefore, we hypothesize that a synthesis of models published in the later phases of the pandemic would produce similar findings. Future research should focus on the later phases of the pandemic, to determine if more models incorporated social determinants of health.

## Conclusion

Our study provides an important overview and synthesis of modelling studies published early in the pandemic. Mathematical models have been, and remain, a critical tool in controlling COVID-19, forecasting the pandemic, estimating the potential impact of policies and interventions, and assessing potential impacts on other conditions. Critical synthesis of modelling studies is an important part of reflective practice, which can lead to improvements in the modelling profession and optimize the usefulness of model-based studies to inform decision-making. We found that few studies incorporated social determinants, and even fewer still incorporated non-age social determinants. The absence of social determinants is concerning for several reasons. Social determinants represent important areas of heterogeneity. Excluding social determinants can reduce modelling accuracy and forego the ability to account for differential uptake, acceptability, affordability, and effectiveness of COVID-19 interventions. Amidst calls to tackle growing health inequities, including social determinants in modelling efforts may provide an important contribution to this effort. Improved data are required to support inclusion of social determinants in models (for example, contact matrices should account for occupational risk, race/ethnicity, household density and income). However, limited availability of data can be partly overcome through modelling techniques. When inclusion of social determinants is a priority for modellers, they can design data collection initiatives to fill in the identified gaps. Modellers should work with stakeholders to formulate research questions reflecting disparities in health and non-health burden, differential access to care, heterogeneity in policy impact and values, and preferences of important social groups. Agent-based models incorporated a greater number of social determinants, but these models also require the most resources. Politicians should fund the infrastructure required to increase modelling complexity and facilitate incorporation of social determinants. Large modelling collaborations can pool intellectual capital and advocate for resources to develop complex models reflecting heterogeneity derived from social determinants. They can also advocate for better data collection efforts, supports for systematic reviews and meta-analyses, and model validation efforts. As countries continue to cope with COVID-19 and prepare for future pandemics, we should take advantage of lessons learned and build a global, coordinated modelling infrastructure that is equipped to account for important social determinants in infectious disease models.

## Data Availability

Study materials, including template data collection forms and extracted data, will be made available upon request.
