# Supplementary material for: Do COVID-19 Infectious Disease Models Incorporate the Social Determinants of Health? A Systematic Review
Source: Public Health Rev. 2024 Oct 10;45:1607057. doi: 10.3389/phrs.2024.1607057 (PMC11499127; doi:10.3389/phrs.2024.1607057)
Supplement: Supplementary file 1 [file DataSheet1.zip › Supplementary File S1.docx]

**Search Strategies**

**Database: Medline August 4, 2020**

**Search Strategy:**

**--------------------------------------------------------------------------------**

1. coronavirus/ or betacoronavirus/ or coronavirus infections/
2. ("coronavirus*" or "coron?virinae*" or "novel coronavirus*" or "novel corona virus" or "Severe Acute Respiratory Syndrome Coronavirus 2" or "coronavirus disease 2019" or "coronavirus pandemic?" or "coronavirus epidemic?" or "coronavirus outbreak?" or "corona virus pandemic?" or "corona virus epidemic?" or "corona virus outbreak?" or "corona virus disease 2019" or "new coronavirus" or "new corona virus*" or "new coronaviruses" or "novel coronaviruses" or "2019 ncov" or "nCov 2019" or "SARS Coronavirus 2" or "2019-nCoV" or "2019nCoV" or "2019-CoV" or "nCoV2019" or "nCoV-2019" or "COVID-19" or "COVID19" or "CORVID-19" or "CORVID19' or WN-CoV or WNCoV'" or "HCoV-19" or "HCoV19" or "CoV " or "2019 novel*" or "Ncov" or "nCov" or "n-cov" or "SARSCoV-2" or "SARSCoV-2" or "SARSCoV2" or "SARS-CoV2" or "SARS-COV-2" or "SARSCov19" or "SARS-Cov19" or "SARSCov-19" or "SARS-Cov-19" or "SARSr-cov" or "Ncovor" or "Ncorona*" or "Ncorono*" or "NcovWuhan*" or "NcovHubei*" or "NcovChina*" or "NcovChinese*" or "Wuhan virus*" or "novel CoV" or "CoV 2" or "CoV2" or "betacoron?vir*").mp.
3. ((corona* or corono*) adj1 (virus* or viral* or virinae*)).mp.
4. (((respiratory* adj2 (acute* or symptom* or disease* or illness* or infect* or condition*)) or "sea-food market*" or "seafood market*" or "food market*" or "foodmarket*" or "wet market*" or "wet-market*" or "wetmarket*") adj10 (Wuhan* or Hubei* or China* or Chinese* or Huanan*)).mp.
5. ((outbreak* or wildlife* or wild-life or pandemic* or epidemic* or coronavirus or corona virus) adj3 (Wuhan* or Hubei* or China* or Chinese* or Huanan*)).mp.
6. (anti-flu* or anti-influenza* or antiflu* or antinfluenza*).mp.
7. or/1-6
8. Models, Theoretical/
9. Models, Biological/
10. Stochastic Processes/ or stochastic.mp.
11. (forecast* or model* or simulat*).af.
12. or/8-11
13. 7 and 12
14. transmi*.ti,kf.
15. 7 and 14
16. 13 or 15
17. limit 16 to yr="2019 -Current"

**Database: Embase Classic+Embase <1947 to 2020 August 07>**

**Search Strategy:**

**--------------------------------------------------------------------------------**

1. coronavirinae/ or betacoronavirus/ or coronavirus infection/
2. ("coronavirus*" or "coron?virinae*" or "novel coronavirus*" or "novel corona virus" or "Severe Acute Respiratory Syndrome Coronavirus 2" or "coronavirus disease 2019" or "coronavirus pandemic?" or "coronavirus epidemic?" or "coronavirus outbreak?" or "corona virus pandemic?" or "corona virus epidemic?" or "corona virus outbreak?" or "corona virus disease 2019" or "new coronavirus" or "new corona virus*" or "new coronaviruses" or "novel coronaviruses" or "2019 ncov" or "nCov 2019" or "SARS Coronavirus 2" or "2019-nCoV" or "2019nCoV" or "2019-CoV" or "nCoV2019" or "nCoV-2019" or "COVID-19" or "COVID19" or "CORVID-19" or "CORVID19' or WN-CoV or WNCoV'" or "HCoV-19" or "HCoV19" or "CoV " or "2019 novel*" or "Ncov" or "nCov" or "n-cov" or "SARSCoV-2" or "SARSCoV-2" or "SARSCoV2" or "SARS-CoV2" or "SARS-COV-2" or "SARSCov19" or "SARS-Cov19" or "SARSCov-19" or "SARS-Cov-19" or "SARSr-cov" or "Ncovor" or "Ncorona*" or "Ncorono*" or "NcovWuhan*" or "NcovHubei*" or "NcovChina*" or "NcovChinese*" or "Wuhan virus*" or "novel CoV" or "CoV 2" or "CoV2" or "betacoron?vir*").mp.
3. ((corona* or corono*) adj1 (virus* or viral* or virinae*)).mp.
4. (((respiratory* adj2 (acute* or symptom* or disease* or illness* or infect* or condition*)) or "sea-food market*" or "seafood market*" or "food market*" or "foodmarket*" or "wet market*" or "wet-market*" or "wetmarket*") adj10 (Wuhan* or Hubei* or China* or Chinese* or Huanan*)).mp.
5. ((outbreak* or wildlife* or wild-life or pandemic* or epidemic* or coronavirus or corona virus) adj3 (Wuhan* or Hubei* or China* or Chinese* or Huanan*)).mp.
6. (anti-flu* or anti-influenza* or antiflu* or antinfluenza*).mp.
7. or/1-6
8. mathematical model/ or compartment model/ or stochastic model/
9. biological model/
10. markov chain/ or stochastic.mp.
11. (forecast* or model* or simulat*).af.
12. or/8-11
13. 7 and 12
14. transmi*.ti,kw.
15. 7 and 14
16. 13 or 15
17. limit 16 to yr="2019 -Current"
18. limit 17 to embase

**Database: EBM Reviews - Cochrane Central Register of Controlled Trials <July 2020>, EBM Reviews - Cochrane Database of Systematic Reviews <2005 to August 07, 2020>**

**Search Strategy:**

**--------------------------------------------------------------------------------**

1. coronavirus/ or betacoronavirus/ or coronavirus infections/
2. ("coronavirus*" or "coron?virinae*" or "novel coronavirus*" or "novel corona virus" or "Severe Acute Respiratory Syndrome Coronavirus 2" or "coronavirus disease 2019" or "coronavirus pandemic?" or "coronavirus epidemic?" or "coronavirus outbreak?" or "corona virus pandemic?" or "corona virus epidemic?" or "corona virus outbreak?" or "corona virus disease 2019" or "new coronavirus" or "new corona virus*" or "new coronaviruses" or "novel coronaviruses" or "2019 ncov" or "nCov 2019" or "SARS Coronavirus 2" or "2019-nCoV" or "2019nCoV" or "2019-CoV" or "nCoV2019" or "nCoV-2019" or "COVID-19" or "COVID19" or "CORVID-19" or "CORVID19' or WN-CoV or WNCoV'" or "HCoV-19" or "HCoV19" or "CoV " or "2019 novel*" or "Ncov" or "nCov" or "n-cov" or "SARSCoV-2" or "SARSCoV-2" or "SARSCoV2" or "SARS-CoV2" or "SARS-COV-2" or "SARSCov19" or "SARS-Cov19" or "SARSCov-19" or "SARS-Cov-19" or "SARSr-cov" or "Ncovor" or "Ncorona*" or "Ncorono*" or "NcovWuhan*" or "NcovHubei*" or "NcovChina*" or "NcovChinese*" or "Wuhan virus*" or "novel CoV" or "CoV 2" or "CoV2" or "betacoron?vir*").mp.
3. ((corona* or corono*) adj1 (virus* or viral* or virinae*)).mp.
4. (((respiratory* adj2 (acute* or symptom* or disease* or illness* or infect* or condition*)) or "sea-food market*" or "seafood market*" or "food market*" or "foodmarket*" or "wet market*" or "wet-market*" or "wetmarket*") adj10 (Wuhan* or Hubei* or China* or Chinese* or Huanan*)).mp.
5. ((outbreak* or wildlife* or wild-life or pandemic* or epidemic* or coronavirus or corona virus) adj3 (Wuhan* or Hubei* or China* or Chinese* or Huanan*)).mp.
6. (anti-flu* or anti-influenza* or antiflu* or antinfluenza*).mp.
7. or/1-6
8. Models, Theoretical/
9. Models, Biological/
10. Stochastic Processes/ or stochastic.mp.
11. (forecast* or model* or simulat*).af.
12. or/8-11
13. 7 and 12
14. transmi*.ti,kw.
15. 7 and 14
16. 13 or 15
17. limit 16 to yr="2019 -Current"

**Database: Web of Science Core Collection 1900 to 2020**

**Search Strategy:**

**--------------------------------------------------------------------------------**

# 1 TS=coronavirus OR TS=betacoronavirus OR TS=coronavirus infections

*Indexes=SCI-EXPANDED, SSCI, A&HCI, CPCI-S, CPCI-SSH, ESCI Timespan=All years*

# 2 KP="coronavirus*" OR KP=“coron?virinae*" OR KP=“novel coronavirus*" OR KP=“novel corona virus" OR KP=“Severe Acute Respiratory Syndrome Coronavirus 2" OR KP=“coronavirus disease 2019" OR KP=“coronavirus pandemic?" OR KP=“coronavirus epidemic?" OR KP=“coronavirus outbreak?" OR KP=“corona virus pandemic?" OR KP=“corona virus epidemic?" OR KP=“corona virus outbreak?" OR KP=“corona virus disease 2019" OR KP=“new coronavirus" OR KP=“new corona virus*" OR KP=“new coronaviruses" OR KP=“novel coronaviruses" OR KP=“2019 ncov" OR KP=“nCov 2019" OR KP=“SARS Coronavirus 2" OR KP=“2019-nCoV" OR KP=“2019nCoV" OR KP=“2019-CoV" OR KP=“nCoV2019" OR KP=“nCoV-2019" OR KP=“COVID-19" OR KP=“COVID19" OR KP=“CORVID-19" OR KP=“CORVID19' OR WN-CoV OR WNCoV'" OR KP=“HCoV-19" OR KP=“HCoV19" OR KP=“CoV KP=“ OR KP=“2019 novel*" OR KP=“Ncov" OR KP=“nCov" OR KP=“n-cov" OR KP=“SARSCoV-2" OR KP=“SARSCoV-2" OR KP=“SARSCoV2" OR KP=“SARS-CoV2" OR KP=“SARS-COV-2" OR KP=“SARSCov19" OR KP=“SARS-Cov19" OR KP=“SARSCov-19" OR KP=“SARS-Cov-19" OR KP=“SARSr-cov" OR KP=“Ncovor" OR KP=“Ncorona*" OR KP=“Ncorono*" OR KP=“NcovWuhan*" OR KP=“NcovHubei*" OR KP=“NcovChina*" OR KP=“NcovChinese*" OR KP=“Wuhan virus*" OR KP=“novel CoV" OR KP=“CoV 2" OR KP=“CoV2" OR KP=“betacoron?vir*"

*Indexes=SCI-EXPANDED, SSCI, A&HCI, CPCI-S, CPCI-SSH, ESCI Timespan=All years*

# 3 KP=((corona* NEAR/1  virus*))

*Indexes=SCI-EXPANDED, SSCI, A&HCI, CPCI-S, CPCI-SSH, ESCI Timespan=All years*

# 4 KP=(anti-flu* or  anti-influenza*  or  antiflu*  or  antinfluenza*)

*Indexes=SCI-EXPANDED, SSCI, A&HCI, CPCI-S, CPCI-SSH, ESCI Timespan=All years*

# 5 #4 OR #3 OR #2 OR #1

*Indexes=SCI-EXPANDED, SSCI, A&HCI, CPCI-S, CPCI-SSH, ESCI Timespan=All years*

# 6 TS=Theoretical Model*

*Indexes=SCI-EXPANDED, SSCI, A&HCI, CPCI-S, CPCI-SSH, ESCI Timespan=All years*

# 7 TS=Mathematical Model*

*Indexes=SCI-EXPANDED, SSCI, A&HCI, CPCI-S, CPCI-SSH, ESCI Timespan=All years*

# 8 TS=Biological Model*

*Indexes=SCI-EXPANDED, SSCI, A&HCI, CPCI-S, CPCI-SSH, ESCI Timespan=All years*

# 9 TS=Stochastic Processes OR KP=stochastic

*Indexes=SCI-EXPANDED, SSCI, A&HCI, CPCI-S, CPCI-SSH, ESCI Timespan=All years*

# 10 KP=(forecast* or  model*  or  simulat*)

Indexes=SCI-EXPANDED, SSCI, A&HCI, CPCI-S, CPCI-SSH, ESCI Timespan=All years

# 11 #10 OR #9 OR #8 OR #7 OR #6

Indexes=SCI-EXPANDED, SSCI, A&HCI, CPCI-S, CPCI-SSH, ESCI Timespan=All years

# 12 #5 AND #11

Indexes=SCI-EXPANDED, SSCI, A&HCI, CPCI-S, CPCI-SSH, ESCI Timespan=All years

# 13 TI=transmi* OR KP=transmi*

Indexes=SCI-EXPANDED, SSCI, A&HCI, CPCI-S, CPCI-SSH, ESCI Timespan=All years

# 14 #5 AND #13

Indexes=SCI-EXPANDED, SSCI, A&HCI, CPCI-S, CPCI-SSH, ESCI Timespan=All years

# 15 #14 OR #12

Indexes=SCI-EXPANDED, SSCI, A&HCI, CPCI-S, CPCI-SSH, ESCI Timespan=All years

# 16 #15

Indexes=SCI-EXPANDED, SSCI, A&HCI, CPCI-S, CPCI-SSH, ESCI Timespan=2019-2020

**Database: medRxiv (January 1, 2019 to August 21, 2020)**

**Search Strategy:**

**--------------------------------------------------------------------------------**

"coronavirus" or "novel coronavirus" or "novel corona virus" or "Severe Acute Respiratory Syndrome Coronavirus 2" or "coronavirus disease 2019" or "coronavirus pandemic" or "coronavirus epidemic" or "coronavirus outbreak" or "corona virus pandemic" or "corona virus epidemic" or "corona virus outbreak" or "corona virus disease 2019" or "new coronavirus" or "new corona virus" or "new coronaviruses" or "novel coronaviruses" or "2019 ncov" or "nCov 2019" or "SARS Coronavirus 2" or "2019-nCoV" or "2019nCoV" or "2019-CoV" or "nCoV2019" or "nCoV-2019" or "COVID-19" or "COVID19" or "CORVID-19" or "CORVID19' or WN-CoV or WNCoV'" or "HCoV-19" or "HCoV19" or "CoV " or "2019 novel" or "Ncov" or "nCov" or "n-cov" or "SARSCoV-2" or "SARSCoV-2" or "SARSCoV2" or "SARS-CoV2" or "SARS-COV-2" or "SARSCov19" or "SARS-Cov19" or "SARSCov-19" or "SARS-Cov-19" or "SARSr-cov" or "Ncovor" or "Ncorona" or "Ncorono" or "NcovWuhan" or "NcovHubei" or "NcovChina" or "NcovChinese" or "Wuhan virus" or "novel CoV" or "CoV 2" or "CoV2" or “anti-flu” or “anti-influenza” or “antiflu” or “antinfluenza”

AND

“stochastic” or “forecast” or “model” or “simulation”

Field:

Abstract or Title
