# Supplementary material for: Do COVID-19 Infectious Disease Models Incorporate the Social Determinants of Health? A Systematic Review
Source: Public Health Rev. 2024 Oct 10;45:1607057. doi: 10.3389/phrs.2024.1607057 (PMC11499127; doi:10.3389/phrs.2024.1607057)
Supplement: Supplementary file 1 [file DataSheet1.zip › Supplementary Table S1.docx]

**Study Characteristics**

**Table S1. Characteristics of COVID-19 modelling studies (Global, 2020)**

|  | **First author** | **Country** | **Setting** | **Target population** | **Goals** | **Model type** | **Main intervention** | **Other interventions** | **Social determinants of health (SDH)** | **Results reported by SDH** | **Goal to assess SDH** |
| --- | --- | --- | --- | --- | --- | --- | --- | --- | --- | --- | --- |
| 1 | An | United States | General population | Children | Impact on other health conditions  Impact of policies or interventions | Simulation | No Intervention | School closure | Sex/Gender  Race/ethnicity | Y | N |
| 2 | Banerjee | England | General population | General population | Predicting the pandemic  Impact on other health conditions | Comparative risk assessment | No Intervention |  | Age  Sex/Gender | Y | Y |
| 3 | Bartsch | United States | General population | General population | Predicting the pandemic  Impact on health services  Economic impact on the population | Simulation | No Intervention |  | Age | Y | N |
| 4 | Borracci | Argentina | General population | General population | Predicting the pandemic  Impact on health services  Impact of policies or interventions | Compartmental | No Intervention | Quarantine (self-isolation)  Lockdown | Age | N | N |
| 5 | Bouchnita | Morocco | General population | General population | Predicting the pandemic  Impact of policies or interventions | Agent-based | No Intervention | Facemasks  Facemasks + Travel restriction | Age | N | N |
| 6 | Britton | Country not specified | General population | General population | Predicting the pandemic  Impact of policies or interventions | Compartmental | No Intervention | Stay at home  Quarantine (self-isolation)  Lockdown | Age | Y | N |
| 7 | Cana-barro | Brazil | General population | General population | Predicting the pandemic  Impact on health services  Impact of policies or interventions | Compartmental | No Intervention | School closure  Physical distancing  Quarantine (self-isolation)  Lockdown | Age | Y | N |
| 8 | Castilho | Brazil | General population | General population | Predicting the pandemic  Impact of policies or interventions | Compartmental | No Intervention | Physical distancing  Quarantine (self-isolation) | Age | Y | Y |
| 9 | Chatterjee | India | General population | General population | Predicting the pandemic  Impact on health services  Impact of policies or interventions | Compartmental | No Intervention | Quarantine (self-isolation) | Age | Y | N |
| 10 | Chikina | United States | General population | General population | Predicting the pandemic  Impact on health services  Impact of policies or interventions | Compartmental | No mitigations at all  optimum homogeneous mitigations (natural relative contact pattern) |  | Age | Y | Y |
| 11 | Chowdhury | Multiple countries | General population | General population | Predicting the pandemic  Impact of policies or interventions  Economic impact on the population | Compartmental | No Intervention | School closure  Stay at home  Physical distancing  Quarantine (self-isolation) | Age  Income | Y | Y |
| 12 | Clark | Multiple countries | General population | General population | Predicting the pandemic | Comparative risk assessment | No Intervention |  | Age  Sex/Gender | Y | Y |
| 13 | Costantino | Australia and China | General population | General population | Predicting the pandemic  Impact of policies or interventions | Compartmental | No Intervention | Travel restriction | Age | Y | Y |
| 14 | Currie | Australia | General population | General population | Predicting the pandemic  Impact on health services  Impact of policies or interventions | Compartmental | No Intervention | Physical distancing  Testing | Age | N | N |
| 15 | Davies a | United Kingdom | General population | General population | Predicting the pandemic  Impact on health services  Impact of policies or interventions | Compartmental | No Intervention | School closure  Stay at home  Physical distancing  Quarantine (self-isolation)  School closure + Stay at home + Physical distancing + Quarantine (self-isolation)  Lockdown | Age | N | N |
| 16 | Davies b | Multiple countries | General population | General population | Predicting the pandemic  Impact of policies or interventions | Compartmental | No Intervention | School closure | Age | Y | Y |
| 17 | Di Domenico | France | General population | General population | Predicting the pandemic  Impact on health services | Compartmental | No Intervention | Physical distancing + School closure + Stay at home + Ban on public gathering | Age | N | Y |
| 18 | Dimeglio | France | General population | General population | Predicting the pandemic  Impact of policies or interventions | Compartmental | No Intervention | Quarantine (self-isolation)  Deconfinement | Age | N | N |
| 19 | Diop | Multiple countries | General population | General population | Predicting the pandemic  Impact of policies or interventions | Compartmental | No Intervention | School closure  Travel restriction International  Travel restriction National  Lockdown  Travel restriction + Lockdown | Age  Remote/rural factors  Urban areas | Y | N |
| 20 | Dizon | Philippines | General population | General population | Predicting the pandemic  Impact of policies or interventions | Compartmental | No Intervention | School closure + Stay at home + Business/economy closure + Ban on public gathering + Physical distancing + Quarantine (self-isolation) + Travel restriction | Age  Occupational factors | Y | N |
| 21 | Djilali | Multiple countries | General population | General population | Predicting the pandemic  Impact of policies or interventions | Compartmental | No Intervention | Quarantine (self-isolation) | Age | N | N |
| 22 | Duczmal | Brazil | General population | General population | Predicting the pandemic  Impact of policies or interventions | Compartmental | No Intervention | Physical distancing Vertical distancing  Physical distancing Horizontal distancing | Age | Y | N |
| 23 | Duque | United States | General population | General population | Impact on health services  Impact of policies or interventions | Compartmental | Lockdown | School closure + Physical distancing + Testing + Contact tracing + Quarantine (self-isolation) + Lockdown | Age | Y | N |
| 24 | Esteve | Multiple countries | General population | General population | Predicting the pandemic  Impact of policies or interventions | Simulation | No Intervention | School closure  Quarantine (self-isolation) | Age  Sex/Gender  Household density | Y | Y |
| 25 | Fan | China | General population  Migrants residing in Wuhan who returned to their hometown | General population | Predicting the pandemic | Simulation | No Intervention |  | Age  Migrants  Remote/rural factors  Occupational factors  Reason for migration  Education  Number of migrating family members per household | Y | N |
| 26 | Gosce | United Kingdom | General population | General population | Predicting the pandemic  Impact of policies or interventions | Compartmental | No Intervention | Testing + Contact tracing + Quarantine (self-isolation) + Facemasks + Lockdown  Testing + Lockdown  Testing + Facemasks + Lockdown  Testing  Testing + Facemasks | Age  Borough level analysis of number of trips by underground and national rail service | Y | Y |
| 27 | Griette | Japan | General population | General population | Predicting the pandemic  Impact of policies or interventions | Compartmental | No Intervention | Physical distancing | Age | Y | Y |
| 28 | Guzzetta | Italy | General population | General population | Predicting the pandemic  Impact on health services | Compartmental | No Intervention |  | Age | Y | N |
| 29 | Hauser | Multiple countries | General population | General population | Predicting the pandemic | Compartmental | No Intervention |  | Age | Y | N |
| 30 | Hilton | Multiple countries | General population | General population | Predicting the pandemic | Compartmental | No Intervention |  | Age | Y | N |
| 31 | Hoertel | France | General population | General population | Predicting the pandemic  Impact on health services  Impact of policies or interventions | Agent-based | No Intervention | Physical distancing + Facemasks  Lockdown | Age | Y | N |
| 32 | Holmes | United States | General population | General population | Predicting the pandemic | Regression | No Intervention |  | Race/ethnicity | Y | Y |
| 33 | Jamieson-Lane | New Zealand | General population | General population | Predicting the pandemic  Impact on health services  Impact of policies or interventions | Compartmental | No Intervention | Lockdown | Age | Y | N |
| 34 | Jewell | Multiple countries | General population | General population | Impact on other health conditions  Impact on health services | Multi-state life table | No Intervention |  | Age  Sex/Gender | N | N |
| 35 | Kamerlin | Sweden | General population | General population | Predicting the pandemic  Impact on health services  Impact of policies or interventions | Agent-based | School closure + Quarantine (self-isolation) | School closure + Quarantine (self-isolation) + Household quarantine  School closure + Business/economy closure + Quarantine (self-isolation)  School closure + Business/economy closure + Physical distancing + Quarantine (self-isolation)  School closure + Stay at home + Quarantine (self-isolation)  School closure + Stay at home + Physical distancing + Quarantine (self-isolation) | Age  Household density | Y | N |
| 36 | Khan | Mauritius | General population | General population | Predicting the pandemic | Auto-regressive | No Intervention |  | Age | N | N |
| 37 | Kim | South Korea | General population | General population | Predicting the pandemic  Impact of policies or interventions | Compartmental | No Intervention | School closure | Age | Y | N |
| 38 | Kirigia | China | General population | General population | Economic impact on the population  Cost-effectiveness of intervention/policies | Multi-state life table | No Intervention |  | Age | Y | Y |
| 39 | Koo | Singapore | General population | General population | Predicting the pandemic  Impact of policies or interventions | Agent-based | No Intervention | School closure + Quarantine (self-isolation)  Physical distancing + Quarantine (self-isolation)  Quarantine (self-isolation)  School closure + Physical distancing + Quarantine (self-isolation) | Age  Sex/Gender  Race/ethnicity  Refugees/Undocumented Workers/Immigrants  Income  Occupational factors  Employment status  Hours worked  Industry  Religion  Marital status  Number of children  Education  Housing type  National service enrollment  Transportation mode  Transportation time  Mobility status | N | N |
| 40 | Kozlovskyi | Multiple countries | General population  Labor migrants | General population  labour migrants | Economic impact on the population | Regression | No Intervention |  | Refugees/Undocumented Workers/Immigrants | Y | Y |
| 41 | Krishnamurthy | India | Urban districts  Public transportation | General population  Commuters | Predicting the pandemic  Impact of policies or interventions | Compartmental | Lockdown |  | Sex/Gender  Commuter density | Y | N |
| 42 | Kucharski | United Kingdom | General population | General population | Predicting the pandemic  Impact of policies or interventions | Simulation | No Intervention | Quarantine (self-isolation)  Quarantine (self-isolation) + Household quarantine  Contact tracing + Quarantine (self-isolation) + Household quarantine  Testing | Age | N | N |
| 43 | Kumar | Multiple countries | General population | General population | Predicting the pandemic | Auto-regressive | No Intervention |  | Literacy rate  Gross Domestic Product (GDP)  (j)Gender Ratio  Rural-Urban ratio  Population density | N | N |
| 44 | Lee | Multiple countries | General population | General population | Predicting the pandemic | Other | No Intervention |  | Age | N | N |
| 45 | Li | China | General population | Patients admitted to the Shandong Provincial Chest Hospital between January 24th and March 16th 2020 | Cost-effectiveness of intervention/policies | Comparative risk assessment | No Intervention | Treatment/Therapy | Age  Sex/Gender  Remote/rural factors | Y | Y |
| 46 | Liu | China | General population | General population | Predicting the pandemic  Impact of policies or interventions | Other | No Intervention | Lockdown | Age  Multigenerational households  Multigenerational workplaces | Y | N |
| 47 | Mahdizadeh Gharakhanlou | Iran | General population | General population | Predicting the pandemic  Impact of policies or interventions | Agent-based | No Intervention | School closure  Business/economy closure  Physical distancing | Age  Household density  Occupational factors  Transportation mode | N | N |
| 48 | Matrajt | United States | General population | General population | Predicting the pandemic  Impact of policies or interventions | Compartmental | No Intervention | Physical distancing | Age | Y | N |
| 49 | Moghadas a | United States | General population | General population | Predicting the pandemic  Impact on health services  Impact of policies or interventions | Compartmental | No Intervention | Quarantine (self-isolation) | Age | Y | N |
| 50 | Moghadas b | United States | General population | General population | Predicting the pandemic  Impact of policies or interventions | Agent-based | No Intervention | Quarantine (self-isolation) | Age | N | N |
| 51 | Mollalo | United States | General population | General population | Predicting the pandemic | Regression | No Intervention |  | Age  Sex/Gender  Race/ethnicity  Income  Access to primary care  Income inequality  Unemployment rate  Air pollution | N | N |
| 52 | Moser | Multiple countries | General population | General population | Predicting the pandemic  Impact on other health conditions | Comparative risk assessment | No Intervention | Stay at home | Age  Sex/Gender  Household density | Y | Y |
| 53 | Ogden | Canada | General population | General population | Predicting the pandemic  Impact on health services  Impact of policies or interventions | Compartmental  Agent-based | No Intervention | School closure  Stay at home  Ban on public gathering  Physical distancing  Contact tracing  Quarantine (self-isolation) | Age | N | N |
| 54 | Pathak | United States | General population  Pediatric hospitalizations due to COVID | Children with COVID-19 requiring hospitalization | Predicting the pandemic  Impact on health services | Comparative risk assessment | No Intervention |  | Age | Y | N |
| 55 | Patrikar | India | General population | General population | Predicting the pandemic  Impact on health services  Impact of policies or interventions | Compartmental | No Intervention | Physical distancing | Age | Y | N |
| 56 | Paul | United States | General population | General population | Predicting the pandemic | Other | No Intervention |  | Age  Race/ethnicity  Remote/rural factors | Y | N |
| 57 | Pourghasemi | Iran | General population | General population | Predicting the pandemic | Auto-regressive | No Intervention |  | Age  Sex/Gender  Distance to public spaces | Y | N |
| 58 | Prem | China | General population | General population | Predicting the pandemic  Impact of policies or interventions | Compartmental | No Intervention |  | Age | Y | N |
| 59 | Puci | Italy | General population | General population | Predicting the pandemic | Compartmental | Travel restriction |  | Age | Y | N |
| 60 | Putra | United States | General population | General population and pregnant females receiving obstetrical care | Predicting the pandemic  Impact on other health conditions  Impact on health services | Other | No Intervention |  | Age  Sex/Gender | N | N |
| 61 | Qiu | China | General population | General population | Predicting the pandemic | Auto-regressive | No Intervention |  | Number of doctors  Population density  GDP per capita | Y | Y |
| 62 | Rainisch | Chile | General population | General population | Predicting the pandemic  Impact on health services  Impact of policies or interventions | Compartmental | No Intervention | School closure + Work from home  Stay at home + Quarantine (self-isolation)  Stay at home + Physical distancing + Quarantine (self-isolation) + Work from home  Quarantine (self-isolation)  Lockdown | Age | N | N |
| 63 | Ren | China | General population | General population | Predicting the pandemic | Regression | No Intervention |  | Floating population  Population density  Bus stops  Subway stations  Length of roads  Rental housing costs  Shopping malls  Supermarkets or convenient stores  Major hospitals  Appointed hospitals or fever clinics | N | N |
| 64 | Ribeiro | Brazil | General population  Remote/Rural community | General population | Predicting the pandemic | Compartmental | No Intervention |  | Remote/rural factors  Indigenous community | N | N |
| 65 | Roberton | Multiple countries | General population | Mothers and children | Impact on health services | Multi-state life table | No Intervention |  | Age | Y | Y |
| 66 | Rost | Hungary | General population | General population | Predicting the pandemic  Impact on health services  Impact of policies or interventions | Compartmental | No Intervention | School closure  Quarantine (self-isolation)  Travel restriction | Age  Population density | Y | N |
| 67 | Salje | France | General population  Hospital | General population | Predicting the pandemic  Impact on health services  Impact of policies or interventions | Compartmental | No Intervention | Lockdown | Age  Sex/Gender | Y | N |
| 68 | Sangiorgio | Italy | Urban districts | General population | Predicting the pandemic  Impact of policies or interventions | Regression | No Intervention | Lockdown | Age  Population density  Industries | N | N |
| 69 | Shoukat | Canada | General population | General population | Predicting the pandemic  Impact on health services  Impact of policies or interventions | Agent-based | No Intervention | Quarantine (self-isolation) | Age | N | N |
| 70 | Sjodin | Italy | Remote/Rural community | General population | Predicting the pandemic  Impact of policies or interventions | Compartmental | No Intervention | Stay at home | Household density | Y | N |
| 71 | Stedman | England | General population | General population | Predicting the pandemic  Impact on health services | Regression | No Intervention |  | Age  Race/ethnicity  Neighbourhood deprivation  Remote/rural factors  Full time work/education  Reported self-confidence in long-term condition management | N | N |
| 72 | Sud a | England | General population | General population | Predicting the pandemic  Impact on other health conditions  Impact on health services | Multi-state life table | No Intervention |  | Age | Y | Y |
| 73 | Sud b | England | General population | Cancer patients | Impact on other health conditions | Multi-state life table | No Intervention |  | Age | Y | N |
| 74 | TrueLove | Bangladesh | Refugee camp | Rohingya refugees in Bangladesh | Predicting the pandemic  Impact on health services | Compartmental | No Intervention |  | Age  Refugees/Undocumented Workers/Immigrants | Y | N |
| 75 | Tuite | Canada | General population | General population | Predicting the pandemic  Impact on health services | Compartmental | Testing + Quarantine (self-isolation) | Testing + Contact tracing  Physical distancing  Physical distancing + Testing + Contact tracing | Age | N | N |
| 76 | Verhagen | England and Wales | General population  Hospital | General population | Predicting the pandemic  Impact on health services | Comparative risk assessment | No Intervention |  | Age  Race/ethnicity  Neighbourhood deprivation  Population density | Y | N |
| 77 | Walker | Multiple countries | General population | General population | Predicting the pandemic  Impact on health services  Impact of policies or interventions | Compartmental | No Intervention | Lockdown | Age  Gross Domestic Product (GDP) | Y | N |
| 78 | Wang | United States | General population | General population | Predicting the pandemic  Impact on health services  Impact of policies or interventions | Compartmental | No Intervention | School closure  School closure + Physical distancing | Age | N | N |
| 79 | Weitz | Country not specified | General population | General population | Predicting the pandemic  Impact on health services  Impact of policies or interventions | Compartmental | No Intervention | Physical distancing | Age | Y | N |
| 80 | Yang | Brazil | General population | General population | Predicting the pandemic  Impact on health services  Impact of policies or interventions | Compartmental | No Intervention | Lockdown | Age | Y | N |
| 81 | Yu a | South Korea | General population | General population | Predicting the pandemic | Other | No Intervention |  | Age  Sex/Gender | Y | Y |
| 82 | Yu b | South Korea | General population | General population | Predicting the pandemic | Auto-regressive | No Intervention |  | Age | Y | Y |
| 83 | Zhao | United States | General population | General population | Predicting the pandemic  Impact on health services  Impact of policies or interventions | Compartmental | Simultaneously releasing all sub-populations from social distancing restrictions |  | Age | Y | N |

References:

1. An, R., Projecting the impact of the coronavirus disease-2019 pandemic on childhood obesity in the United States: A microsimulation model. Journal of sport and health science, 2020. 9(4): p. 302-312.

2. Banerjee, A., et al., Estimating excess 1-year mortality associated with the COVID-19 pandemic according to underlying conditions and age: a population-based cohort study. Lancet (London, England), 2020. 395(10238): p. 1715-1725.

3. Bartsch, S.M., et al., The Potential Health Care Costs And Resource Use Associated With COVID-19 In The United States. Health affairs (Project Hope), 2020. 39(6): p. 927-935.

4. Borracci, R.A. and N.D. Giglio, Forecasting the effect of social distancing on COVID-19 autumn-winter outbreak in the metropolitan area of Buenos Aires. Estimacion del efecto del distanciamiento social sobre la epidemia de COVID-19 de otono-invierno en el area metropolitana de Buenos Aires., 2020. 80 Suppl 3: p. 7-15.

5. Bouchnita, A. and A. Jebrane, A multi-scale model quantifies the impact of limited movement of the population and mandatory wearing of face masks in containing the COVID-19 epidemic in Morocco. Math. Model. Nat. Phenom., 2020. 15: p. 13.

6. Britton, T., F. Ball, and P. Trapman, A mathematical model reveals the influence of population heterogeneity on herd immunity to SARS-CoV-2. Science (New York, N.Y.), 2020.

7. Canabarro, A., et al., Data-driven study of the COVID-19 pandemic via age-structured modelling and prediction of the health system failure in Brazil amid diverse intervention strategies. PloS one, 2020. 15(7): p. e0236310.

8. Castilho, C., et al., ASSESSING THE EFFICIENCY OF DIFFERENT CONTROL STRATEGIES FOR THE COVID-19 EPIDEMIC. Electron. J. Differ. Equ., 2020: p. 17.

9. Chatterjee, K., A. Kumar, and S. Shankar, Healthcare impact of COVID-19 epidemic in India: A stochastic mathematical model. Medical journal, Armed Forces India, 2020.

10. Chikina, M. and W. Pegden, Modeling strict age-targeted mitigation strategies for COVID-19. PloS one, 2020. 15(7): p. e0236237.

11. Chowdhury, R., et al., Dynamic interventions to control COVID-19 pandemic: a multivariate prediction modelling study comparing 16 worldwide countries. European journal of epidemiology, 2020. 35(5): p. 389-399.

12. Clark, A., et al., Global, regional, and national estimates of the population at increased risk of severe COVID-19 due to underlying health conditions in 2020: a modelling study. The Lancet. Global health, 2020. 8(8): p. e1003-e1017.

13. Costantino, V., D.J. Heslop, and C.R. MacIntyre, The effectiveness of full and partial travel bans against COVID-19 spread in Australia for travellers from China during and after the epidemic peak in China. Journal of travel medicine, 2020.

14. Currie, D.J., et al., Stemming the flow: how much can the Australian smartphone app help to control COVID-19? Public health research & practice, 2020. 30(2).

15. Davies ^b^, N.G., et al., Age-dependent effects in the transmission and control of COVID-19 epidemics. Nature medicine, 2020. 26(8): p. 1205-1211.

16. Davies ^a^, N.G., et al., Effects of non-pharmaceutical interventions on COVID-19 cases, deaths, and demand for hospital services in the UK: a modelling study. The Lancet. Public health, 2020. 5(7): p. e375-e385.

17. Di Domenico, L., et al., Impact of lockdown on COVID-19 epidemic in Ile-de-France and possible exit strategies. BMC medicine, 2020. 18(1): p. 240.

18. Dimeglio, C., et al., The SARS-CoV-2 seroprevalence is the key factor for deconfinement in France. The Journal of infection, 2020. 81(2): p. 318-356.

19. Diop, B.Z., et al., The relatively young and rural population may limit the spread and severity of COVID-19 in Africa: a modelling study. BMJ global health, 2020. 5(5).

20. Dizon, R.L., The heterogeneous age-mixing model of estimating the covid cases of different local government units in the National Capital Region, Philippines. Clinical Epidemiology and Global Health, 2020.

21. Djilali, S. and B. Ghanbari, Coronavirus pandemic: A predictive analysis of the peak outbreak epidemic in South Africa, Turkey, and Brazil. Chaos, solitons, and fractals, 2020. 138: p. 109971.

22. Duczmal, L.H., et al., Vertical social distancing policy is ineffective to contain the COVID-19 pandemic. Cadernos de saude publica, 2020. 36(5): p. e00084420.

23. Duque, D., et al., Timing social distancing to avert unmanageable COVID-19 hospital surges. Proceedings of the National Academy of Sciences of the United States of America, 2020.

24. Esteve, A., et al., National age and coresidence patterns shape COVID-19 vulnerability. Proceedings of the National Academy of Sciences of the United States of America, 2020. 117(28): p. 16118-16120.

25. Fan, C., et al., Prediction of Epidemic Spread of the 2019 Novel Coronavirus Driven by Spring Festival Transportation in China: A Population-Based Study. International journal of environmental research and public health, 2020. 17(5).

26. Gosce, L., et al., Modelling SARS-COV2 Spread in London: Approaches to Lift the Lockdown. The Journal of infection, 2020. 81(2): p. 260-265.

27. Griette, Q., P. Magal, and O. Seydi, Unreported Cases for Age Dependent COVID-19 Outbreak in Japan. Biology, 2020. 9(6).

28. Guzzetta, G., et al., Potential short-term outcome of an uncontrolled COVID-19 epidemic in Lombardy, Italy, February to March 2020. Euro surveillance : bulletin Europeen sur les maladies transmissibles = European communicable disease bulletin, 2020. 25(12).

29. Hauser, A., et al., Estimation of SARS-CoV-2 mortality during the early stages of an epidemic: A modeling study in Hubei, China, and six regions in Europe. PLoS medicine, 2020. 17(7): p. e1003189.

30. Hilton, J. and M.J. Keeling, Estimation of country-level basic reproductive ratios for novel Coronavirus (SARS-CoV-2/COVID-19) using synthetic contact matrices. PLoS computational biology, 2020. 16(7): p. e1008031.

31. Hoertel, N., et al., A stochastic agent-based model of the SARS-CoV-2 epidemic in France. Nature medicine, 2020.

32. Holmes, L., Jr., et al., Black-White Risk Differentials in COVID-19 (SARS-COV2) Transmission, Mortality and Case Fatality in the United States: Translational Epidemiologic Perspective and Challenges. International journal of environmental research and public health, 2020. 17(12).

33. Jamieson-Lane, A. and E. Cytrynbaum, Effects of age-targeted sequestration for COVID-19. Journal of biological dynamics, 2020. 14(1): p. 621-632.

34. Jewell, B.L., et al., Potential effects of disruption to HIV programmes in sub-Saharan Africa caused by COVID-19: results from multiple mathematical models. The lancet. HIV, 2020.

35. Kamerlin, S.C.L. and P.M. Kasson, Managing COVID-19 spread with voluntary public-health measures: Sweden as a case study for pandemic control. Clinical infectious diseases : an official publication of the Infectious Diseases Society of America, 2020.

36. Khan, S. and A. Alfaifi, Modeling of Coronavirus Behavior to Predict it's Spread. Int. J. Adv. Comput. Sci. Appl., 2020. 11(5): p. 394-399.

37. Kim, S., et al., School Opening Delay Effect on Transmission Dynamics of Coronavirus Disease 2019 in Korea: Based on Mathematical Modeling and Simulation Study. Journal of Korean medical science, 2020. 35(13): p. e143.

38. Kirigia, J.M. and R.N.D.K. Muthuri, The fiscal value of human lives lost from coronavirus disease (COVID-19) in China. BMC research notes, 2020. 13(1): p. 198.

39. Koo, J.R., et al., Interventions to mitigate early spread of SARS-CoV-2 in Singapore: a modelling study. The Lancet. Infectious diseases, 2020. 20(6): p. 678-688.

40. Kozlovskyi, S., et al., The system dynamic model of the labor migrant policy in economic growth affected by COVID-19. Glob. J. Environ. Sci. Manag., 2020. 6: p. 95-106.

41. Krishnamurthy, K., et al., Prediction of the transition from sub-exponential to the exponential transmission of SARS-CoV-2 and epidemic nowcasting for metro-zones: Experiences from Chennai-Metro-Merge, India. JMIR public health and surveillance, 2020.

42. Kucharski, A.J., et al., Effectiveness of isolation, testing, contact tracing, and physical distancing on reducing transmission of SARS-CoV-2 in different settings: a mathematical modelling study. The Lancet. Infectious diseases, 2020.

43. Kumar, A., et al., Data-driven modelling and prediction of COVID-19 infection in India and correlation analysis of the virus transmission with socio-economic factors. Diabetes & metabolic syndrome, 2020. 14(5): p. 1231-1240.

44. Lee, S.Y., B. Lei, and B. Mallick, Estimation of COVID-19 spread curves integrating global data and borrowing information. PloS one, 2020. 15(7): p. e0236860.

45. Li, X.Z., et al., Treatment of coronavirus disease 2019 in Shandong, China: a cost and affordability analysis. Infectious diseases of poverty, 2020. 9(1): p. 78.

46. Liu, Y., et al., What are the Underlying Transmission Patterns of COVID-19 Outbreak? - An Age-specific Social Contact Characterization. EClinicalMedicine, 2020: p. 100354.

47. Mahdizadeh Gharakhanlou, N. and N. Hooshangi, Spatio-temporal simulation of the novel coronavirus (COVID-19) outbreak using the agent-based modeling approach (case study: Urmia, Iran). Informatics in Medicine Unlocked, 2020. 20: p. 100403.

48. Matrajt, L. and T. Leung, Evaluating the Effectiveness of Social Distancing Interventions to Delay or Flatten the Epidemic Curve of Coronavirus Disease. Emerging infectious diseases, 2020. 26(8): p. 1740-1748.

49. Moghadas ^b^, S.M., et al., The implications of silent transmission for the control of COVID-19 outbreaks. Proceedings of the National Academy of Sciences of the United States of America, 2020. 117(30): p. 17513-17515.

50. Moghadas ^a^, S.M., et al., Projecting hospital utilization during the COVID-19 outbreaks in the United States. Proceedings of the National Academy of Sciences of the United States of America, 2020. 117(16): p. 9122-9126.

51. Mollalo, A., K.M. Rivera, and B. Vahedi, Artificial Neural Network Modeling of Novel Coronavirus (COVID-19) Incidence Rates across the Continental United States. International journal of environmental research and public health, 2020. 17(12).

52. Moser, D.A., et al., Years of life lost due to the psychosocial consequences of COVID-19 mitigation strategies based on Swiss data. European psychiatry : the journal of the Association of European Psychiatrists, 2020. 63(1): p. e58.

53. Ogden, N.H., et al., Modelling scenarios of the epidemic of COVID-19 in Canada. Canada communicable disease report = Releve des maladies transmissibles au Canada, 2020. 46(8): p. 198-204.

54. Pathak, E.B., et al., COVID-19 in Children in the United States: Intensive Care Admissions, Estimated Total Infected, and Projected Numbers of Severe Pediatric Cases in 2020. Journal of public health management and practice : JPHMP, 2020. 26(4): p. 325-333.

55. Patrikar, S., et al., Projections for novel coronavirus (COVID-19) and evaluation of epidemic response strategies for India. Medical journal, Armed Forces India, 2020. 76(3): p. 268-275.

56. Paul, R., et al., Progression of COVID-19 From Urban to Rural Areas in the United States: A Spatiotemporal Analysis of Prevalence Rates. The Journal of rural health : official journal of the American Rural Health Association and the National Rural Health Care Association, 2020.

57. Pourghasemi, H.R., et al., Spatial modeling, risk mapping, change detection, and outbreak trend analysis of coronavirus (COVID-19) in Iran (days between February 19 and June 14, 2020). International journal of infectious diseases : IJID : official publication of the International Society for Infectious Diseases, 2020. 98: p. 90-108.

58. Prem, K., et al., The effect of control strategies to reduce social mixing on outcomes of the COVID-19 epidemic in Wuhan, China: a modelling study. The Lancet. Public health, 2020. 5(5): p. e261-e270.

59. Puci, M.V., et al., COVID-19 Trend Estimation in the Elderly Italian Region of Sardinia. Frontiers in public health, 2020. 8: p. 153.

60. Putra, M., et al., Forecasting the impact of coronavirus disease during delivery hospitalization: an aid for resource utilization. American Journal of Obstetrics and Gynecology MFM, 2020: p. 100127.

61. Qiu, Y., X. Chen, and W. Shi, Impacts of social and economic factors on the transmission of coronavirus disease 2019 (COVID-19) in China. Journal of population economics, 2020: p. 1-46.

62. Rainisch, G., E.A. Undurraga, and G. Chowell, A dynamic modeling tool for estimating healthcare demand from the COVID19 epidemic and evaluating population-wide interventions. International journal of infectious diseases : IJID : official publication of the International Society for Infectious Diseases, 2020. 96: p. 376-383.

63. Ren, H., et al., Early forecasting of the potential risk zones of COVID-19 in China's megacities. The Science of the total environment, 2020. 729: p. 138995.

64. Ribeiro, S.P., et al., Severe airport sanitarian control could slow down the spreading of COVID-19 pandemics in Brazil. PeerJ, 2020. 8: p. e9446.

65. Roberton, T., et al., Early estimates of the indirect effects of the COVID-19 pandemic on maternal and child mortality in low-income and middle-income countries: a modelling study. The Lancet. Global health, 2020. 8(7): p. e901-e908.

66. Rost, G., et al., Early Phase of the COVID-19 Outbreak in Hungary and Post-Lockdown Scenarios. Viruses, 2020. 12(7).

67. Salje, H., et al., Estimating the burden of SARS-CoV-2 in France. Science (New York, N.Y.), 2020. 369(6500): p. 208-211.

68. Sangiorgio, V. and F. Parisi, A multicriteria approach for risk assessment of Covid-19 in urban district lockdown. Safety science, 2020. 130: p. 104862.

69. Shoukat, A., et al., Projecting demand for critical care beds during COVID-19 outbreaks in Canada. CMAJ : Canadian Medical Association journal = journal de l'Association medicale canadienne, 2020. 192(19): p. E489-E496.

70. Sjodin, H., et al., Only strict quarantine measures can curb the coronavirus disease (COVID-19) outbreak in Italy, 2020. Eurosurveillance, 2020. 25(13).

71. Stedman, M., et al., COVID-19: Generate and apply local modelled transmission and morbidity effects to provide an estimate of the variation in overall relative healthcare resource impact at general practice granularity. International journal of clinical practice, 2020: p. e13533.

72. Sud ^b^, A., et al., Collateral damage: the impact on outcomes from cancer surgery of the COVID-19 pandemic. Annals of oncology : official journal of the European Society for Medical Oncology, 2020. 31(8): p. 1065-1074.

73. Sud ^a^, A., et al., Effect of delays in the 2-week-wait cancer referral pathway during the COVID-19 pandemic on cancer survival in the UK: a modelling study. The Lancet. Oncology, 2020. 21(8): p. 1035-1044.

74. Truelove, S., et al., The potential impact of COVID-19 in refugee camps in Bangladesh and beyond: A modeling study. PLoS medicine, 2020. 17(6): p. e1003144.

75. Tuite, A.R., D.N. Fisman, and A.L. Greer, Mathematical modelling of COVID-19 transmission and mitigation strategies in the population of Ontario, Canada. CMAJ : Canadian Medical Association journal = journal de l'Association medicale canadienne, 2020. 192(19): p. E497-E505.

76. Verhagen, M.D., et al., Forecasting spatial, socioeconomic and demographic variation in COVID-19 health care demand in England and Wales. BMC medicine, 2020. 18(1): p. 203.

77. Walker, P.G.T., et al., The impact of COVID-19 and strategies for mitigation and suppression in low- And middle-income countries. Science, 2020. 369(6502): p. 413-422.

78. Wang, X., et al., Impact of Social Distancing Measures on Coronavirus Disease Healthcare Demand, Central Texas, USA. Emerging infectious diseases, 2020. 26(10).

79. Weitz, J.S., et al., Modeling shield immunity to reduce COVID-19 epidemic spread. Nature medicine, 2020. 26(6): p. 849-854.

80. Yang, H.M., et al., Mathematical model describing CoViD-19 in Sao Paulo, Brazil - evaluating isolation as control mechanism and forecasting epidemiological scenarios of release. Epidemiology and infection, 2020. 148: p. e155.

81. Yu ^b^, X., Risk Interactions of Coronavirus Infection across Age Groups after the Peak of COVID-19 Epidemic. International journal of environmental research and public health, 2020. 17(14).

82. Yu ^a^, X., et al., Distinctive trajectories of the COVID-19 epidemic by age and gender: A retrospective modeling of the epidemic in South Korea. International journal of infectious diseases : IJID : official publication of the International Society for Infectious Diseases, 2020. 98: p. 200-205.

83. Zhao, H. and Z. Feng, Staggered release policies for COVID-19 control: Costs and benefits of relaxing restrictions by age and risk. Mathematical biosciences, 2020. 326: p. 108405.
